# Potentiating Vγ9Vδ2 T cell proliferation and assessing their cytotoxicity towards adherent cancer cells at the single cell level

**DOI:** 10.1242/bio.059049

**Published:** 2022-02-07

**Authors:** Chenxiao Liu, Karolina Skorupinska-Tudek, Sven-Göran Eriksson, Ingela Parmryd

**Affiliations:** 1 Science for Life Laboratory, Department of Medical Cell Biology, Uppsala University, 751 23 Uppsala, Sweden; 2 Laboratory of Lipid Biochemistry, Institute of Biochemistry and Biophysics, Polish Academy of Sciences, 02-106 Warsaw, Poland; 3 Department of Biomedicine, The Sahlgrenska Academy, University of Gothenburg, 405 30 Gothenburg, Sweden

**Keywords:** Colon cancer, Cytotoxicity assay, Phosphoantigens, Proliferation, Vγ9Vδ2 T cells

## Abstract

Vγ9Vδ2 T cells is the dominant γδ T cell subset in human blood. They are cytotoxic and activated by phosphoantigens whose concentrations are increased in cancer cells, making the cancer cells targets for Vγ9Vδ2 T cell immunotherapy. For successful immunotherapy, it is important both to characterise Vγ9Vδ2 T cell proliferation and optimise the assessment of their cytotoxic potential, which is the aim of this study. We found that supplementation with freshly thawed human serum potentiated Vγ9Vδ2 T cell proliferation from peripheral mononuclear cells (PBMCs) stimulated with (*E*)-4-Hydroxy-3-methyl-but-2-enyl diphosphate (HMBPP) and consistently enabled Vγ9Vδ2 T cell proliferation from cryopreserved PBMCs. In cryopreserved PBMCs the proliferation was higher than in freshly prepared PBMCs. In a panel of short-chain prenyl alcohols, monophosphates and diphosphates, most diphosphates and also dimethylallyl monophosphate stimulated Vγ9Vδ2 T cell proliferation. We developed a method where the cytotoxicity of Vγ9Vδ2 T cells towards adherent cells is assessed at the single cell level using flow cytometry, which gives more clear-cut results than the traditional bulk release assays. Moreover, we found that HMBPP enhances the Vγ9Vδ2 T cell cytotoxicity towards colon cancer cells. In summary, we have developed an easily interpretable method to assess the cytotoxicity of Vγ9Vδ2 T cells towards adherent cells, found that Vγ9Vδ2 T cell proliferation can be potentiated by media-supplementation and how misclassification of non-responders may be avoided. Our findings will be useful in the further development of Vγ9Vδ2 T cell immunotherapy.

## Abstract (precis)

**Summary:** HMBPP potentiated Vγ9Vδ2 T cell cytotoxicity towards colon cancer cells, assessed at the single cell level using a novel flow cytometric method. Dimethylallyl monophosphate is a phosphoantigen and freshly thawed serum supplementation improves proliferation.

## INTRODUCTION

Vγ9Vδ2 T cells are found in primates and are part of the innate immune system (Morita et al., 2007) and also function as a bridge between the innate and adaptive immune systems since they possess antigen presenting capacity towards both CD4+ and CD8+ αβ T cells (Brandes et al., 2005; 2009). Vγ9Vδ2 T cells comprise about 3-5% of the normal circulating T cell population (Andreu-Ballester et al., 2012; Tan et al., 2016), but after a week's infection can increase to 60% of total circulating T cells (Morita et al., 2007). Vγ9Vδ2 T cells are activated by small non-peptide compounds, phosphoantigens, of which the most potent, (*E*)-4-hydroxy-3-methyl-but-2-enyl diphosphate (HMBPP), is produced in the 2-*C*-methyl-D-erythritol 4-phosphate pathway for isoprenoid synthesis (Hintz et al., 2001; Jomaa et al., 1999). This pathway is present in plants and many pathogens but is absent in humans, who instead use the mevalonate pathway for isoprenoid synthesis. In the mevalonate pathway, the intermediate isopentenyl diphosphate (IPP) is a well-known phosphoantigen (Tanaka et al., 1995).

Phosphoantigens, released by for instance *P. falciparum* infected erythrocytes (Liu et al., 2018), activate Vγ9Vδ2 T cells via interactions with the intracellular B30.2 domain (Sandstrom et al., 2014; Wang and Morita, 2015) of the almost ubiquitously expressed protein CD277/butyrophilin-3A1 (BTN3A1) (Harly et al., 2012). Upon phosphoantigen interaction the BTN3A1 undergoes a conformational change that is sensed by the Vγ9Vδ2 T cells (Gu et al., 2017; Yang et al., 2019). The release of phosphoantigens by erytrhocytes requires that they must cross the plasma membrane of BTN3A1-expressing cells for activation to occur, which is an energy-dependent process (Kilcollins et al., 2016).

Cancer cells frequently have an upregulated mevalonate pathway due to increased metabolic demands (Gottesman et al., 2002; Gruenbacher and Thurnher, 2015), which causes the accumulation of IPP and other isoprenoid diphosphates that can activate and cause the proliferation of Vγ9Vδ2 T cells (Gruenbacher et al., 2014). The subsequent infiltration of Vγ9Vδ2 T cells in the tumours results in tumour immunosurveillance (Fowler and Bodman-Smith, 2015) and Vγ9Vδ2 T cells have been shown to kill a broad range of cancer cells (Angelini et al., 2004; Das et al., 2001; Gertner et al., 2007; Wrobel et al., 2007). Inhibitors of the mevalonate-pathway enzyme farnesyl diphosphate synthase, aminobisphosphonates (van Beek et al., 1999) and alkylamines (Thompson et al., 2006), that lead to the intracellular accumulation of the phosphoantigen IPP also lead to the activation of Vγ9Vδ2 T cells.

Circulating Vγ9Vδ2 T cells in cancer patients are less abundant and/or less responsive than those in healthy individuals (Gaafar et al., 2009; Petrini et al., 2011; Puan et al., 2009; Saitoh et al., 2008; Toia et al., 2016; Wilhelm et al., 2003). This suggests that the Vγ9Vδ2 T cells are important in the protection against cancer. It is currently not known whether the Vγ9Vδ2 T cell numbers decrease as result of the disease or whether individuals with low fractions of circulating Vγ9Vδ2 T cells are predisposed to developing cancer. However, there seems to be no correlation between the circulating percentage of Vγ9Vδ2 T cells in PBMCs and their ability to proliferate in the presence of phosphoantigens (Cabillic et al., 2010), making Vγ9Vδ2 T cells promising candidates for immunotherapy.

To assess the cytotoxicity of Vγ9Vδ2 T cells towards adherent target cells, which includes most types of cancer cells, the target cells are usually labelled, commonly with ^51^Cr (Capietto et al., 2011; Fisher et al., 2014b; Wrobel et al., 2007). Cytotoxicity is then monitored by the appearance of label in the media. Besides the hazard of working with a radiolabel, this type of bulk assay fails to differentiate between leakage from live cells and release from dead cells. Therefore, the same numerical response could originate from widely different combinations of live, damaged and dead cells. For target cells that grow in suspension, FACS-based cytotoxicity methods are frequently used (Cosan et al., 2017; Jedema et al., 2004; Lecoeur et al., 2001; Sawaisorn et al., 2019), but for adherent target cells this approach is rare. FACS-based cytotoxicity assays are based on the differential staining of mostly intact dead versus live cells, but is unable to account for cells that disintegrate. Thus, there is a general need for more informative cytotoxicity tests, particularly for adherent target cells.

In this study, we have developed a flow cytometry-based method that reports cell death at the single cell level, to assess the cytotoxicity of Vγ9Vδ2 T cells towards adherent cells. We have also addressed how DMSO affects Vγ9Vδ2 T cell proliferation capacity and addressed how Vγ9Vδ2 T cells in PBMCs proliferate in response to isoprenoid alcohols, monophosphates and diphosphates. Moreover, we found that reactivation increases Vγ9Vδ2 T cell cytotoxicity towards colon cancer cells and found a crucial role for supplementation of freshly thawed serum in Vγ9Vδ2 T cell proliferation.

## RESULTS

### Cryopreservation of PBMCs enhances the Vγ9Vδ2 T cell proliferative response

**Fig. 1.**
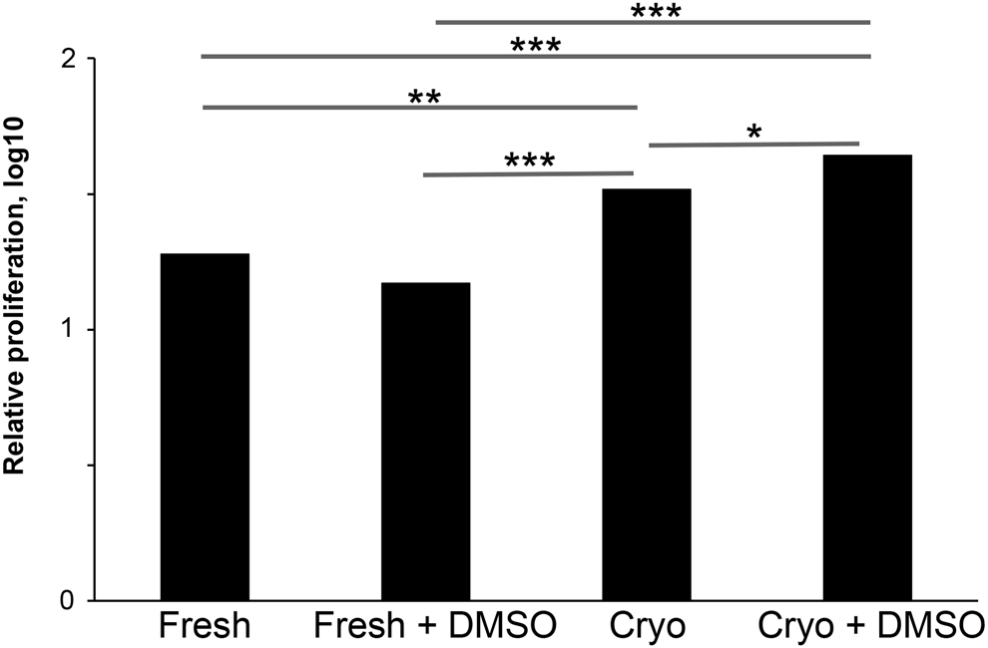
**The effect of DMSO on Vδ2 T cell proliferation.** Freshly isolated and cryopreserved PBMCs were stimulated with 80 pM HMBPP±0.1% DMSO. 25 U/ml IL-2 was added on days 3, 5 and 7. Proliferation was assessed on days 11-12. For the statistical analysis, the data were transformed to the log10-scale. A linear statistical model based on data from 11 donors with freshly prepared or cryopreserved samples, with or without DMSO, donors and the experimental date, were used as explanatory factors. The response variable in the model was relative proliferation after 10-logarithmic transformation. Relative proliferation was defined as the difference between the absolute number of Vδ2 T cells at the end and the start of the experiment with the proliferation of the negative control (IL-2 only) subtracted. Data shown are effects from different treatments. *P=1.2×10^−2^, **P=0.0071 and ***P=<0.001 for a two-tailed *t*-test.

In Vγ9Vδ2 T cell immunotherapy clinical trials, multiple cell infusions of expanded cells are performed (Fisher et al., 2014a), and repeated expansion from the same PBMC batch could prove advantageous. Hence, being able to preserve cells without loss of function is desirable. To assess whether cryopreservation affected the extent of Vγ9Vδ2 T cell proliferation, freshly prepared and cryopreserved PBMCs from the same donors were compared using a linear statistical model encompassing data from 11 donors. From cryopreserved PBMCs, stimulated with the phosphoantigen HMBPP and lymphocyte growth stimulatory interleukin IL-2, Vγ9Vδ2 T cells proliferated from all donors tested. Interestingly, the Vγ9Vδ2 T cell proliferation from cryopreserved PBMCs was actually higher than that from freshly prepared PBMCs from the same donors (Fig. 1). Cryopreservation resulted in substantial overall cell loss (about 40%), but the fraction of Vδ2+CD3+ lymphocytes was not significantly different in freshly prepared and cryopreserved PBMCs from the same donors, suggesting that variability in survival is not the cause of the difference in proliferation (data not shown).

### DMSO enhances the phosphoantigen-induced proliferation of cryopreserved Vγ9Vδ2 T cells

Our finding that Vγ9Vδ2 T cells could be more efficiently proliferated from cryopreserved PBMCs than freshly prepared PBMCs prompted us the investigate the underlying reason. Although the cryopreserved PBMCs were washed twice after thawing, it seemed possible that some of the DMSO, used at 10% (vol) during cryopreservation, remained in the media after washing the cells and/or was excreted into the media after the cells had been thawed. We therefore tested whether DMSO had any effect on Vγ9Vδ2 T cell proliferation stimulated by HMBPP, using the same linear model as above. Interestingly, the presence of 0.1% DMSO enhanced the Vγ9Vδ2 T cell proliferation from cryopreserved PBMCs, but it did not result in any statistically significant effect on proliferation from freshly prepared PBMCs (Fig. 1). As expected, a higher DMSO concentration (2.5%) killed both the freshly prepared and cryopreserved PBMCs (data not shown).

### Freshly thawed serum supplementation is important for Vγ9Vδ2 T cell proliferation

**Fig. 2.**
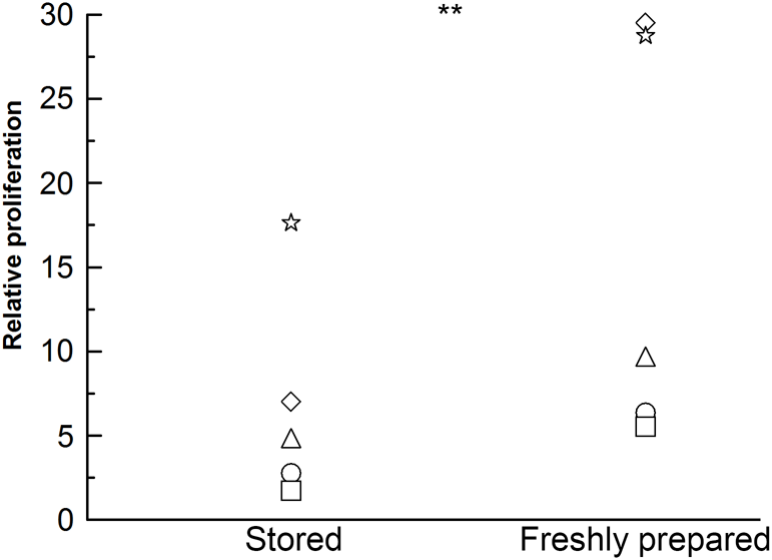
**Freshly added serum improves Vδ2 T cell proliferation.** Cryopreserved PBMCs were seeded at 1×10^6^ cells/ml and stimulated with 80 pM HMBPP. 25 U/ml IL-2 was added on days 3, 5 and 7. On day 0 and after 11 days, the PBMCs and the stimulated cultures were assessed for their CD3 and Vδ2 expression by flow cytometry analysis. Relative proliferation is defined as the difference in the absolute number of Vδ2 T cells at the end and the start of the experiment with the proliferation of the negative control (IL-2 only) subtracted. The data shown are the means of two biological replicates. Fresh covers the supplementation of freshly thawed heat-inactivated human AB serum and glutamine just before use. Stored covers media with human AB serum added 17 days prior to the start of the experiment stored at 4°C with freshly thawed glutamine added just before use. The data were transformed to the log10-scale for the statistical analysis. **P=0.0055 for a two-tailed paired *t*-test, *n*=5.

It is well known that primary cells are more sensitive to growth conditions than most cultured cells and that the glutamate in cell media is quickly degraded, but the importance of freshly thawed serum for Vγ9Vδ2 T cell proliferation has, to our knowledge, not been considered. We therefore assessed the effect of freshly thawed serum on the proliferation of Vγ9Vδ2 T cells. Complete medium that had been stored for 17 days at 4°C was compared with freshly made complete medium. Glutamine was added to both media at the start of the experiment since it is known to decompose upon storage of media (Heeneman et al., 1993). Proliferation stimulated by HMBPP and IL-2 was consistently greater with the media where freshly thawed serum was supplemented at the start of the experiment (Fig. 2) indicating that some serum components are quickly lost during storage at 4°C. After proliferation with HMBPP, the population of Vγ9Vδ2 T cells were mainly of central and effector memory phenotypes (not shown).

Previously, we noticed that when using stored complete medium (up to 6 months old), Vγ9Vδ2 T cells from healthy donors aged 20-65 years occasionally failed to proliferate from PBMCs, but after switching to using medium supplemented with freshly thawed serum Vγ9Vδ2 T cell proliferation occurred with all healthy donors (data not shown). Our data demonstrate that the use of stored medium can reduce the Vγ9Vδ2 T cell proliferative response and even cause the misclassification of responders as non-responders.

### Vγ9Vδ2 T cells proliferate in response to a range of isoprenoid diphosphates length C5-C20 and the monophosphate DMAP

**Fig. 3.**
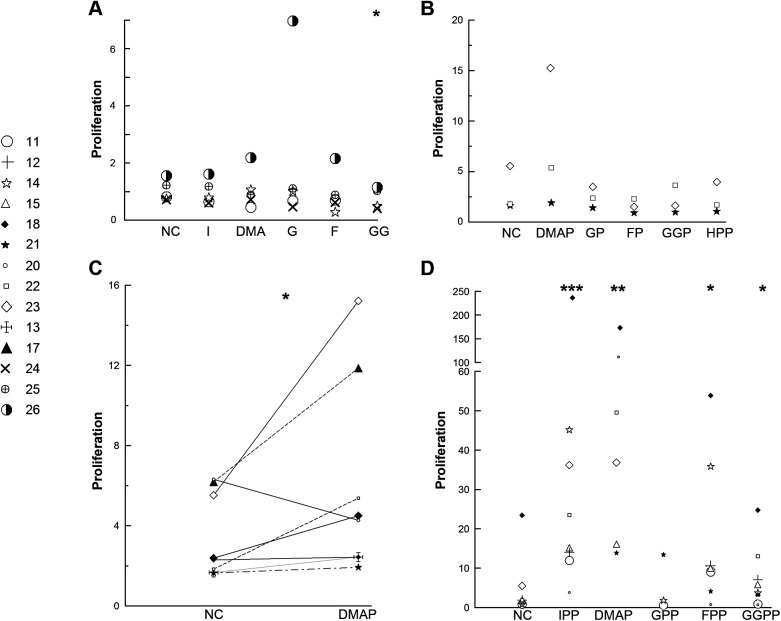
**Proliferation of Vδ2 T cells from PBMCs stimulated by isoprenoid alcohols, mono- and diphosphates.** (A) Cryopreserved PBMCs were seeded at 1×10^6^ live cells/ml and stimulated with 25 μM isopentenol (I), dimethylallyl alcohol (DMA), geraniol (G), farnesol (F) or geranylgeraniol (GG). (B) Freshly isolated PBMCs were seeded at 1×10^6^ live cells/ml and stimulated with 25 μM DMAP, GP, FP, GGP or heptaprenyl phosphate (HPP). (C) Cryopreserved PBMCs were seeded at 1×10^6^ live cells/ml and stimulated with 25 μM DMAP. (D) Freshly isolated or cryopreserved PBMCs were seeded at 1×10^6^ live cells/ml and stimulated with 25 μM IPP, DMAPP, GPP, FPP or GGPP. 25 U/ml IL-2 was added on days 3, 5 and 7. On day 0 and after 11-12 days, the PBMCs and the stimulated cultures were assessed for their CD3 and Vδ2 expression by flow cytometry. Proliferation is defined as the difference between the absolute number of Vδ2 T cells at the end and the start of the experiment and the data shown are the means of two biological replicates. NC=negative control (IL-2 only). The data were transformed to the log10-scale for the statistical analysis. *P<0.05, **P<0.01 and ***P<0.001 for a one-tailed paired *t*-test, *n*=3-8.

Whether isoprenoid monophosphates and alcohols can stimulate Vγ9Vδ2 T cell proliferation from PBMCs has, to be best of our knowledge, not been systematically addressed. Isoprenoid alcohols do not qualify as phosphoantigens per se, but they can be taken up by cells and used for protein prenylation (Parmryd et al., 1999), strongly suggesting that they are converted intracellularly into their diphosphate counterparts, i.e. phosphoantigens. However, at 25 µM none of the isoprenoid alcohols, isopentenol (C5), dimethylallyl alcohol (DMA, C5), geraniol (G, C10) or farnesol (F, C15) were able to stimulate Vγ9Vδ2 T cell proliferation (Fig. 3A). Varying the concentration of DMA from 0.25–250 µM did not alter this conclusion (data not shown). In two experiments, the longer alcohols geranylgeraniol (GG, C20) and heptaprenol (HP, C35) were also unable to cause Vγ9Vδ2 T cell proliferation.

Isoprenoid monophosphates are genuine phosphoantigens, i.e. small non-peptide antigens with phosphate groups (Burk et al., 1995; Gossman and Oldfield, 2002). Out of a panel of five isoprenoid monophosphates, the only one that stimulated a proliferation response at 25 µM was dimethylallyl monophosphate (DMAP) (Fig. 3B). This was examined in a larger group of donors where a proliferation response was seen in seven out of eight donors (Fig. 3C).

Until recently, the single isoprenoid unit diphosphate IPP was considered to outperform its longer counterparts in activating Vγ9Vδ2 T cells (Morita et al., 2007). However, the study used Vγ9Vδ2 T cell clones and clones appear to respond differently to phosphoantigen stimulation than PBMCs, whose stimulation with diphosphates one to four isoprenoid units long have been reported to be comparable (Gruenbacher et al., 2014). Upon testing a panel of isoprenoid diphosphates for their ability to stimulate Vγ9Vδ2 T cell proliferation from PBMCs, we found that 25 µM IPP, DMAPP (dimethyl allyl diphosphate), FPP (farnesyl diphosphate) and GGPP (geranylgeranyl diphosphate) were all effective (Fig. 3D). Geranyl diphosphate (GPP) showed the same tendency but did not reach statistical significance (*P*=0.17), possibly because of the concentration chosen since substantial proliferation was observed with GPP at 56 µM (*P*=0.0027, *N*=2). In two experiments, heptaprenyl diphosphate (HPPP) did not stimulate Vγ9Vδ2 T cell proliferation.

### Vγ9Vδ2 T cell cytotoxicity towards adherent colon cancer cells can be measured using flow cytometry

To study the cytotoxicity of Vγ9Vδ2 T cells against adherent cells, methods that measure the bulk release, typically of ^51^Cr or CFSE, from the target cells are usually employed. Unfortunately, these methods are neither very precise nor accurate. We therefore set out to develop a flow cytometry method for measuring Vγ9Vδ2 T cell cytotoxicity towards adherent target cells, a method that reports the absolute number of dead cells. Routinely, large effector cell numbers are used in cytotoxicity assays, but the limited number of Vγ9Vδ2 cells in small volume patient samples is a constraint. We therefore developed a protocol for small numbers of effector cells, using polypropylene 96-well round bottom plates. Assays in FACS tubes typically require a volume of at least 200 µl, but in the wells we were able to use only 50 µl. The number of target cells used was limited to 2500, which were incubated with the desired number of effector cells.

For the flow cytometry analysis to accurately report on the number of target cells killed, it requires that all the target cells are in suspension at the end of the incubation. However, the target cells may firmly adhere to the surface in which the cytotoxicity assay takes place. By microscopy, we confirmed that SW620 cells remained attached at the bottom of the wells after suspension with a 100 µl pipette after a 4 h incubation at 37°C (Fig. S1). The same was found with HT29, SW420 and Colo205 cells (not shown).

**Fig. 4.**
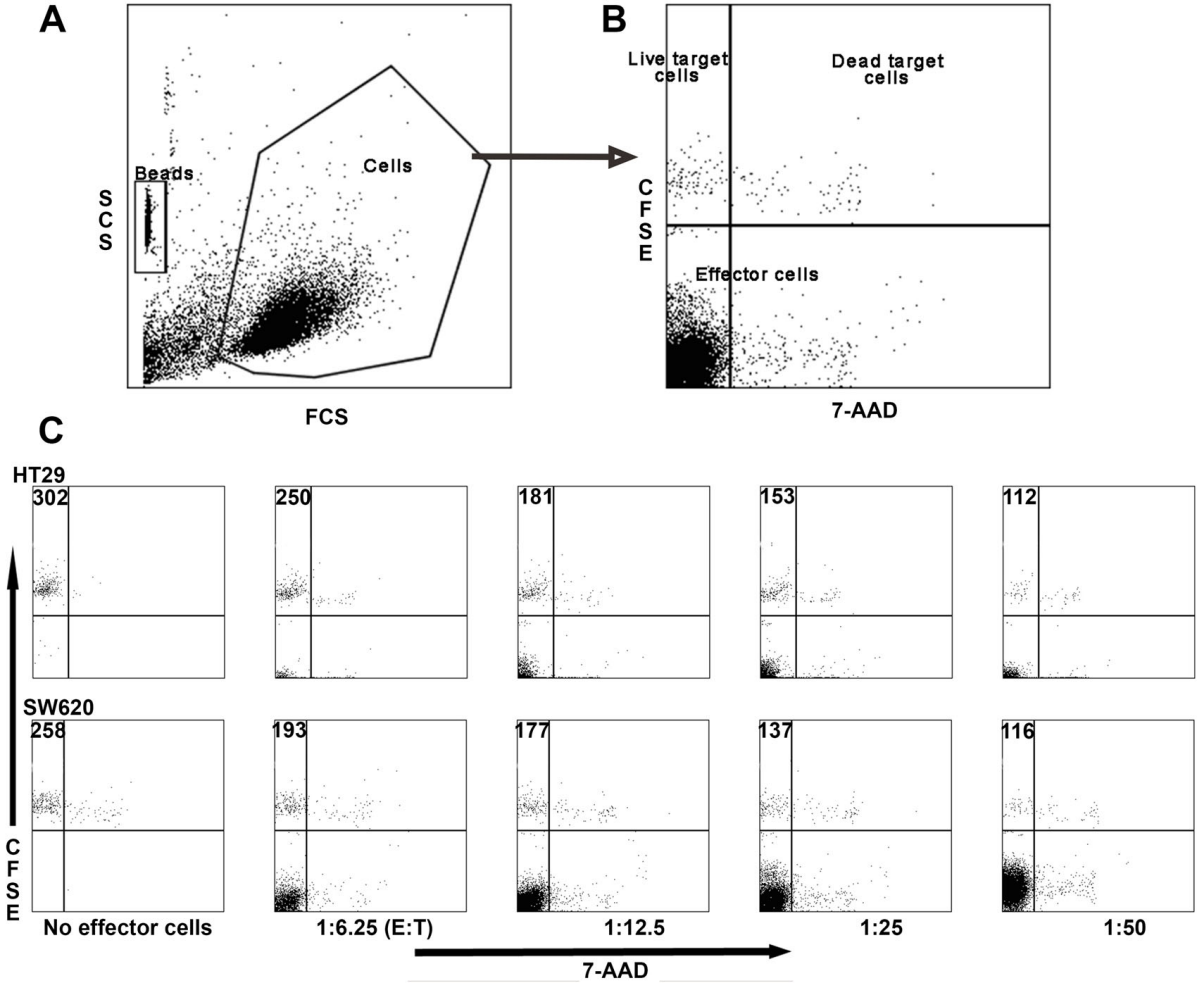
**Flow cytometric analysis of colon cancer cell killing by *in vitro* expanded Vγ9Vδ2 T cells.** Adherent target cells were detached with trypsin-EDTA, washed and stained with CFSE. Vγ9Vδ2 T effector cells were obtained by stimulating PBMCs with 80 pM HMBPP and adding of IL-2 (25 U/ml) every second day, starting on day 3. The expanded γδ T cells were purified by negative selection using magnetic beads after 12 days in culture. Purified effector cells were incubated in 25 U IL-2/ml overnight, after which they were incubated with target cells for 4 h at 37°C. Beads were added as a counting reference and the dead cells were stained with 7-AAD. The cells were gated using the side and forward scatter dot plot (A). Note that most 7-AAD positive cells do not appear in the gated population since dead cells have altered forward and side scatter compared with the gated population. Live target cells were CFSE+ but 7AAD- (top left quadrant) and dying target cells were double positive (top right quadrant). (B) The effector cell populations are found in the lower left and right quadrants. (C) The effector to target cell ratio was varied between 6.25:1 and 50:1 and compared with a target-cell-free control. The number of live target cells is displayed.

**Fig. 5.**
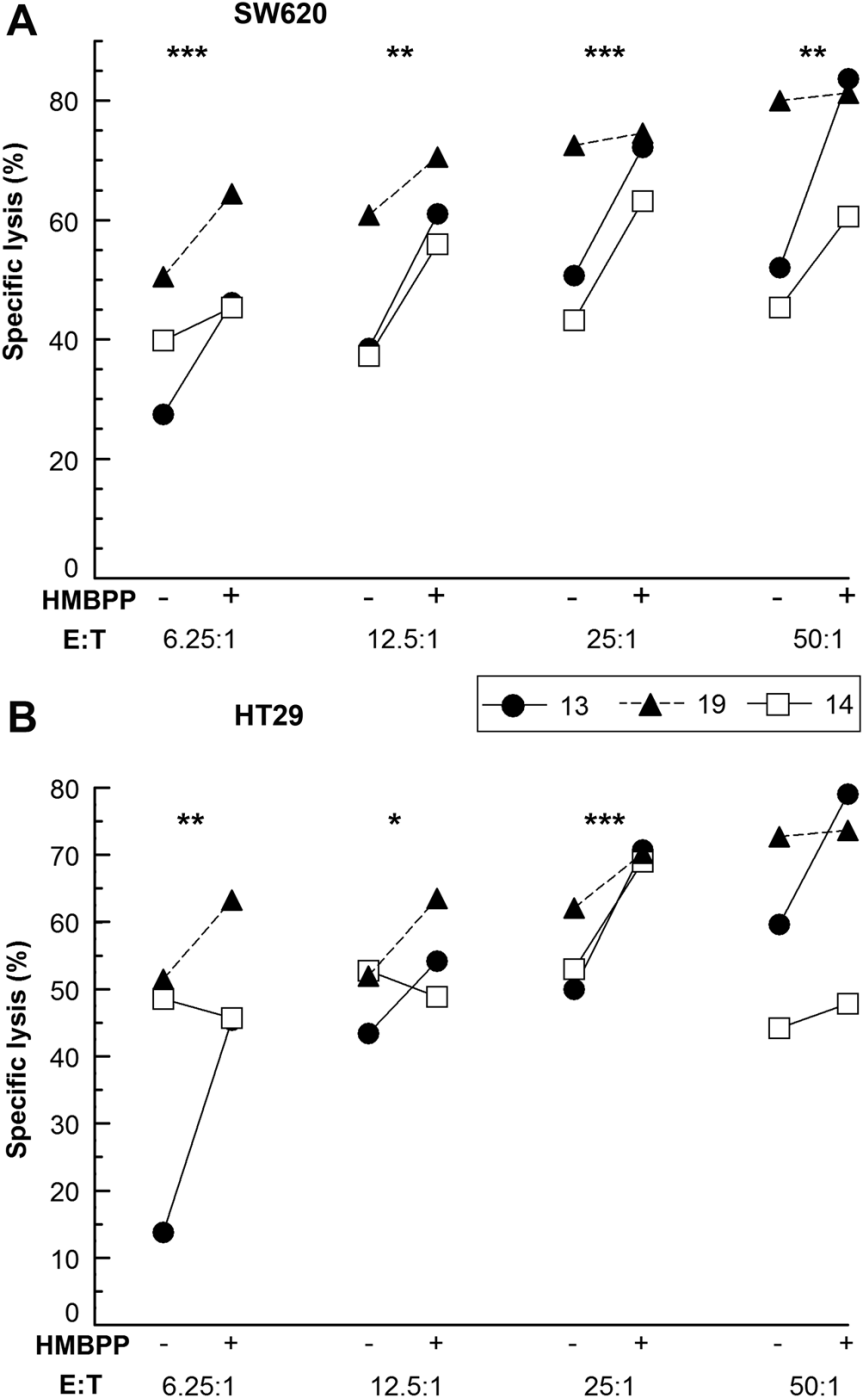
**HMBPP enhances the cytotoxicity of *in vitro* expanded Vγ9Vδ2 T cells towards colon cancer cells.** PBMCs, freshly prepared or cryopreserved, were seeded at 1×10^6^ live cells/ml and stimulated with 80 pM HMBPP. IL-2 (25 U/ml) was added every second day starting on day 3. Total γδ cells (effector cells, E) were purified by negative selection using magnetic beads after 11-12 days of culture. The purified effector cells (E) were incubated with (A) SW620 and (B) HT29 target (T) cells±80 pM HMBPP at different E:T ratios for 4 h at 37°C. Specific lysis was determined by flow cytometric analysis. The data shown are means from three biological replicates and stars indicate the statistical significance from an ANOVA for each E:T ratio.

Flow cytometry-based cytotoxicity methods that rely on assessing the number of live and/or stained dead cells at the end of an experiment understate the cytotoxicity, since cells that disintegrate are not registered. To circumvent this problem, a constant number of microbeads was added to each sample as a counting reference (Fig. 4A). The target cells were identified using CFSE and 7-AAD staining to differentiate between live and dying/dead cells (Fig. 4B). Note that most 7-AAD positive cells do not appear in the gated population since dead cells have altered forward and side scatter compared with the gated population. An illustration of the FACS output of our assay is found in Fig. 5C. By incrementally increasing the number of effector Vγ9Vδ2 T cells, a corresponding decrease in live target cells in the upper left quadrant can be seen. As the effector to target ratio is increased, the increase of effector cells is evident in the lower left quadrant.

### HMBPP improves the cytotoxicity of Vγ9Vδ2 T cells towards adherent colon cancer cells

Phosphoantigens have been reported to increase the cytotoxicity of Vγ9Vδ2 T cells when used for restimulation before and/or during the mixing of the effector cells with suspension target cells (Dunne et al., 2010). We wanted to address whether this also holds for adherent target cells and therefore assessed the Vγ9Vδ2 T cell cytotoxicity towards colon cancer-derived cell lines HT29 (stage II-III) and SW620 (stage III) in the presence of HMBPP at a range of effector to target cell ratios. Across the whole range of E:T ratios from 6.25:1 to 50:1, the presence of HMBPP enhanced the cytotoxicity of Vγ9Vδ2 T cell towards SW620 cells and for HT29 cells and an enhancing effect was found for the ratios 6.25:1 to 25:1 (Fig. 5). For SW620 cells, the effect of HMBPP stimulation of Vγ9Vδ2 T cell cytotoxicity was also assessed at lower E:T ratios and found to have a potentiating effect at all ratios tested including one as low as 1:1 (Fig. S2). To assess the accuracy of the Vγ9Vδ2 T cell flow cytometry cytotoxicity method, 95% confidence intervals for the fraction of dead cells were calculated for mean values obtained from three biological replicates (Table 1). The size of the intervals was similar for all effector to target cell ratios.

**Table 1.**
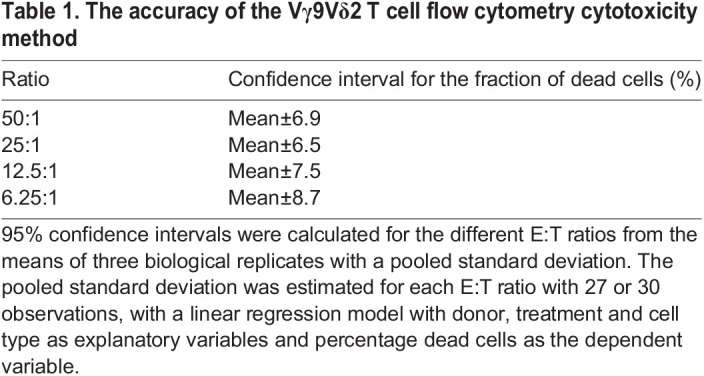
The accuracy of the Vγ9Vδ2 T cell flow cytometry cytotoxicity method

## DISCUSSION

Samples are rarely collected synchronously from individual donors, but it is advantageous to characterise and proliferate Vγ9Vδ2 T cells from multiple donors concurrently, both for efficiency and comparability. Hence being able to cryopreserve cells without loss of function is desirable. We found that the Vγ9Vδ2 T cells that survived cryopreservation had a greater proliferative capacity than Vγ9Vδ2 T cells in freshly prepared PBMCs from the same donor. This differs from a previous study that found that proliferation of Vγ9Vδ2 T cells is considerably lower in cryopreserved PBMCs than freshly prepared samples (Siegers et al., 2012). The discrepancy may be attributed to differential media supplementation with freshly thawed serum, which we found was an important requirement for efficient proliferation of Vγ9Vδ2 T cells. Interestingly, DMSO increased the proliferation from the cryopreserved PBMCs, but not from freshly prepared PBMCs, suggesting that freezing and thawing may make cells more susceptible to this treatment. Other supplements reported to increase Vγ9Vδ2 T cell proliferation include IL-15, vitamin C and its more stable phosphorylated derivative (Kouakanou et al., 2020; Van Acker et al., 2016). A combination of these supplements may lead to even more efficient Vγ9Vδ2 T cell proliferation of importance for immunotherapy approaches.

We did not find any difference in the percentage of Vδ2+ T cells in freshly prepared and cryopreserved PBMCs from the same donors, but still it is possible that a minor population of Tregs that restrict Vγ9Vδ2 T cell proliferation are specifically eliminated by cryopreservation. The higher Vγ9Vδ2 T cell proliferation from the cryopreserved PBMCs may also simply reflect that only the fittest cells survive thawing and freezing.

Vγ9Vδ2 T cells can kill a broad range of cancer cells and show potential for use in immunotherapy with several clinical trials showing promise (Cabillic et al., 2010; Nakajima et al., 2010; Wada et al., 2014). It has been shown that the radiosensitivity of γδ T cells does not differ from that of αβ T cells (Lisowska et al., 2010), which is important since combined cancer therapy is showing encouraging results (Loskog et al., 2016; Mangsbo et al., 2010; Todaro et al., 2013a). However, in the presence of IL-21, Vγ9Vδ2 T cells can develop into immunosuppressive Tregs (Barjon et al., 2017), emphasising that both the expansion conditions and the tumour microenvironment can influence the outcome of adoptive Vγ9Vδ2 T cell transfer. Long-term *in vitro* expansion of Vγ9Vδ2 T cells may also lead to activation-induced cell death, which can be reduced using vitamin C supplementation or antigen presenting cells expression ligands to CD80, CD83, 4-1BB, CD32, CD36, CD40 L and CD40 (Cho et al., 2015; Choi et al., 2021; Kouakanou et al., 2020).

For Vγ9Vδ2 T cell immunotherapy by adoptive transfer of *ex vivo* expanded cells to be considered, it is critical that the patients’ cells respond to stimulation and thus can be expanded. Our finding that supplementing media freshly thawed serum prevents the misclassification of responders as non-responders, argues that using freshly prepared medium should become the standard protocol for assessing Vγ9Vδ2 T cell proliferation. Possibly in combination with thawing cryopreserved PBMCs in human albumin that was recently shown to improve Vγ9Vδ2 T cell survival upon thawing (Burnham et al., 2021). The omission of freshly thawed serum supplementation may explain the surprisingly large fraction of non-responders reported in some studies (Petrini et al., 2011; Wilhelm et al., 2003), especially since the non-responders were present in the control group, whereas we found only responders among healthy donors after switching to medium supplemented with freshly thawed serum.

It was long believed that single isoprenoid unit diphosphates IPP and DMAPP were superior at activating Vγ9Vδ2 T cells than their longer counterparts (Morita et al., 2007). However, it was later suggested that this only applies to Vγ9Vδ2 T cell clones. When PBMCs were used as the starting material, isoprenoid diphosphates one to four isoprenoid units long resulted in comparable levels of proliferation (Gruenbacher et al., 2014). Our results corroborate this finding, but unlike Gruenbacher et al., we needed a higher GPP concentration to observe proliferation comparable to that of PBMCs stimulated by IPP, DMAPP, FPP and GGPP. This difference could be the result of us using a lower, more physiologically relevant, IL-2 concentration (Pullman and Doe, 1992), as well as our use of a more quantitative proliferation assay. Further emphasising the importance of the starting material, in Vγ9Vδ2 T cell clones, IPP is more potent than DMAPP (Morita et al., 2001; 2007), whereas the opposite is true for PBMCs (Amslinger et al., 2007).

Whether the longer isoprenoid disphosphates, like their shorter counterparts IPP and HMBPP, can bind to and cause a conformation change of BTN3A1 is unclear (Vavassori et al., 2013). An alternative to binding BTN3A1is that they like aminobisphosphonates and alkylamines are indirect phosphoantigens that inhibit farnesyl diphosphate synthase, which leads to intracellular IPP accumulation.

We found that DMAP, but not the longer isoprenoid monophosphates, stimulated Vγ9Vδ2 T cell proliferation from PBMCs at the same concentration as DMAPP. (*E*)-4-hydroxy-3-methyl-but-2-enyl phosphate (HMBP) from the 2-*C*-methyl-D-erythritol 4-phosphate pathway, has also been reported to support Vγ9Vδ2 T cell proliferation from PBMCs, but with a potency about 1700 times lower than that of HMBPP, making the difference between the mono- and diphosphate bigger than for DMAP/DMAPP (Amslinger et al., 2007). In Vγ9Vδ2 T cell clones, DMAP was found to be considerably more potent in stimulating proliferation than isopentenyl phosphate (IP), whereas the reverse was found for the corresponding disphosphates (Morita et al., 2001; Tanaka et al., 1995). Intriguingly isoprenoid monophosphates, unlike their diphosphate counterparts, are resistant to dephosphorylation by the ECTO-ATPase CD39 (Gruenbacher et al., 2016) and they therefore could be stable activators of Vγ9Vδ2 T cells *in vivo*.

Isoprenoid alcohols are taken up by cells, probably more readily than their phosphorylated counterparts, and are incorporated into prenylated proteins by enzymes requiring phosphorylated substrates (Crick et al., 1997; Parmryd et al., 1999), i.e. they are converted to phosphoantigens intracellularly. Given that phosphoantigens are believed to be sensed intracellularly by the B30.2 domain of BTN3A1 (Sandstrom et al., 2014; Wang and Morita, 2015), it is surprising that the isoprenoid alcohols did not stimulate Vγ9Vδ2 T cell proliferation. A possible explanation is that the phosphorylation of the alcohols does not occur in the compartment containing BTN3A1. However, protein prenylation takes place in the cytosol (Moores et al., 1991; Reiss et al., 1990; Yoshida et al., 1991), where BTN3A1 exposes its intracellular domain, so there could be an enzyme complex that channels the phosphorylated alcohols directly to the protein prenylation substrates, precluding their exposure to BTN3A1. Alternatively, higher concentrations of isoprenoid pyrophosphates may be required to achieve a conformation change of BTN3A1 than are required for protein prenylation. The concentration of phosphoantigen required to produce a conformational change of BTN3A1 remains to be established.

Despite the hazards of working with radiochemicals ^51^Cr-release assays are routinely used to assess cytotoxicity towards adherent target cells (Capietto et al., 2011; Fisher et al., 2014b; Wrobel et al., 2007). Replacing ^51^Cr with a non-radioactive label like CFSE eliminates this hazard, but neither approach can differentiate between live and dead cells. This means that the same quantitative response could have a range of biological explanations: a few cells being completely ruptured, many cells becoming slightly leaky or a mix of ruptured and leaky cells. For cells that grow in suspension, multiple FACS analysis methods for the assessment of cytotoxicity exist (Jedema et al., 2004; Kim et al., 2007; Sawaisorn et al., 2019), but for adherent cells a FACS approach is less common. We have demonstrated that FACS is also an excellent methodology for assessing Vγ9Vδ2 T cell cytotoxicity towards adherent cells. Our method has two major advantages: it requires few cells, and it allows the exact quantification of the number of dead cells, including cells that disintegrate, by using beads as a counting reference. Accordingly, our method is a major improvement upon the bulk-release methods.

We found that HMBPP enhanced the cytotoxicity of Vγ9Vδ2 T cells towards adherent colon cancer cells, which is in line with earlier findings with suspension target cells (Dunne et al., 2010). The most likely explanation for the enhancement is that HMBPP by activating the Vγ9Vδ2 T cells stimulate the release of granula containing IFN-γ and/or granylysin/perforin, both of which would promote cytotoxicity. The expansion protocol is also known to affect the cytotoxicity of Vγ9Vδ2 T cells. Aminobisphonates, which cause the accumulation of phosphoantigens, leads to the proliferation of Vγ9Vδ2 T cells with a cytotoxic phenotype when administered to colon cancer stem cells used to stimulate PBMCs *in vitro* (Zocchi et al., 2017). Chemotherapy has also been shown to prime cancer cells to killing by of Vγ9Vδ2 T cells (Todaro et al., 2013b). Injection with aminobisphoshonates is also used in clinical trials in combination with IL-2 to induce the *in vivo* proliferation of cytotoxic Vγ9Vδ2 T cells (Dieli et al., 2003). Adoptive cell transfer of *in vitro* expanded Vγ9Vδ2 T cells may be a preferable approach however, since injections of aminobisphonates have been reported to be associated with the onset and/or worsening of inflammatory and autoimmune disorders (Markovits et al., 2017). A promising approach is targeting Vγ9Vδ2 T cells to cancer cells using bispecific antibodies (Oberg et al., 2014). To optimise the proliferation as well as the cytotoxicity of Vγ9Vδ2 T cells, a combination of these approaches may prove beneficial.

In conclusion, using our novel, quantitative flow cytometry method, the cytotoxicity of Vγ9Vδ2 T cells towards adherent cells can be assessed with a straightforward interpretation at the single cell level. The crucial role for supplementation of freshly thawed serum when assessing Vγ9Vδ2 T cell proliferation that we present will help to minimise misclassification as non-responders, which is important for testing patients for compatibility with Vγ9Vδ2 T cell immunotherapy.

## MATERIALS AND METHODS

### Materials

RPMI 1640, DMEM, glutamine, EDTA-Trypsin (0.25%) and penicillin/streptomycin were from GE Healthcare HyClone (Logan, UT, USA). Human AB serum (HS) was from Lonza (Basel, Switzerland) or the blood centre at Uppsala University Hospital, Sweden. The antibodies anti-Vδ2-FITC (clone B6), anti-CD3-PE (clone UCHT1), anti-mouse IgG-PE (clone MOPC-21) and 7-amino-actinomycin D (7-AAD) were from BioLegend (San Diego, CA, USA). CFSE was from Invitrogen (Thermo Fisher Scientific, Waltham, MA, USA). Fetal bovine serum (FBS), Histopaque^®^-1077, isopentenol, *trans*-geraniol, all-*trans*-farnesol, dimethylallyl alcohol for isoprenoid phosphate synthesis and chemicals were from Sigma (St. Louis, MO, USA), unless otherwise indicated. HPLC-grade solvents were from Merck (Darmstadt, Germany). HMBPP was from Echelon Biosciences (Salt Lake City, UT, USA), dimethylallyl alcohol was from Isoprenoids (Tampa, FL) and all-*trans*-geranylgeraniol was from Santa Cruz Biotechnology (Dallas, TX). Heptaprenol was isolated from the wood of *Betula verrucosa* (Chojnacki et al., 1975). The IL-2 was a generous gift from Giampetro Corradin (Université de Lausanne, Switzerland).

### Ethical statement

This study was conducted in accordance with the principles expressed in the Declaration of Helsinki and was approved by the Ethical Review Boards in Stockholm (2007/823-31/2) and Uppsala (2011/850-32).

### Culture of cell lines

Both SW620 cells and HT29 cells were from ATCC, the latter via Dr. A. Blokzijl, Uppsala University. Both cell lines were cultured in DMEM supplemented with 10% FBS, 100 U/ml penicillin, 100 µg/ml streptomycin and 2 mM glutamine (full media). The cell cultures tested negative for Mycoplasma infection, assessed using a PCR-based method (Uphoff and Drexler, 2011).

### Synthesis of isoprenoid mono- and diphosphates

Dimethylallyl and isopentenyl mono- and diphosphates were synthesised at the Collection of Polyprenols, Institution of Biochemistry and Biophysics (IBB), Polish Academy of Science (PAS) according to the large-scale method for allylic isoprenoid diphosphates (Davisson et al., 1985). The synthesis of all other isoprenoid mono- and diphosphates was performed at the Collection of Polyprenols, IBB, PAS according to the polyprenyl phosphate synthesis method (Danilov et al., 1989). Stock solutions of isoprenoids were kept in 0.15 M NH_3_:EtOH (1:1, v/v).

### PBMC preparation

Buffy coats from healthy donors were obtained from the Blood Central at Uppsala University Hospital. Peripheral blood mononuclear cells (PBMCs) were prepared using Histopaque^®^-1077. The PBMCs were washed twice in ice-cold RPMI-1640. For cryopreservation at −80°C, the PBMCs were suspended at 10-20×10^6^/ml in RPMI-1640 freshly supplemented with 10% human AB-serum, 100 U/ml penicillin, 100 µg/ml streptomycin and 2 mM glutamine and 10% DMSO. The PBMCs were stored at −80°C for periods up to 8 months.

### Vγ9Vδ2 T cell proliferation

Cryopreserved PBMCs were thawed quickly in a water bath at 37°C and washed twice in RPMI-1640. Both cryopreserved and freshly isolated PBMCs were suspended at 1×10^6^ live cells/ml in RPMI-1640 supplemented with 5% human AB-serum, 100 U/ml penicillin, 100 µg/ml streptomycin, 25 mM HEPES and 2 mM glutamine (complete medium). All isoprenoids except HMBPP were added from 1.4-25 mM stocks in NH_4_:EtOH (1:1) to the bottom of wells in 24-well plates. HMBPP was added from a freshly prepared 80 nM solution in NH_4_:EtOH (1:1). The solvent was allowed to evaporate before cells at 1×10^6^/ml in complete RPMI-1640 were added. The final HMBPP concentration was 80 pM and the final concentrations of all other isoprenoids was 25 µM. DMSO (0.1-2.5%) was added to the culture vials before the addition of cells where indicated. The cells were cultured at 37°C in 5% CO_2_ in a humidified atmosphere (LabRum, Stockholm, Sweden). At days 3, 5 and 7, 25 U/ml IL-2 was added when proliferation was to be assessed. IL-2 was also added on day 9, when the cells were assessed for their cytotoxicity. On day 7, 200 µl complete medium was added to all samples. The cells were harvested on days 10-11.

### Purification of γδ T cells

The expanded γδ T cells were isolated using a negative selection TCR γ/δ+ T cell isolation kit according to the instructions of the manufacturer (Miltenyi Biotech GmbH, Gladbach, Germany). The purified cells were incubated at 1×10^6^/ml in complete media supplemented with 25 U/ml IL-2 at 37°C in 5% CO_2_ in a humidified atmosphere overnight. The purified cells were assessed by flow cytometry and contained ≥90% Vδ2 T cells.

### Cytotoxicity assay

SW620 and HT29 cells in T25 flasks were washed in PBS and incubated with 1 ml EDTA-Trypsin for 5 min at 37°C. The detached cells were counted using a Countess Automated Cell Counter (Invitrogen, Carlsbad, CA, USA) and suspended at 1×10^6^/ml in full media. The target cells were labelled with 2 µM CFSE in PBS at 37°C in the dark for 20-25 min, after which they were washed once in 10 mL PBS and suspended at 1×10^5^/ml in full media. 25µL of the target cell suspension was added per well of 96-well polystyrene plates (VWR, Radnor, PA, USA). The effector Vδ2+ T cells were washed once, resuspended in complete media at 5×10^6^/ml and added to the wells at effector to target cells ratios of 50:1, 25:1, 12.5:1 and 6.25:1 or 27:1, 9:1, 3:1 and 1:1. The total volume was adjusted to 50 µl in all wells and the cell mixes were incubated at 37°C for 4 h. The cell mixtures were then transferred to Falcon round-bottom polystyrene tubes (Corning, NY) and kept on ice. 0.125 µg 7-AAD from a 50 µg/ml stock in PBS with sodium azide [final concentration 0.00075% (w/v)] and a constant volume (containing around 20,000) of Calibrite beads (BD Biosciences, San Jose, CA) were added to each tube. The samples were analysed by flow cytometry after a minimum of 5 and maximum of 60 min on ice. The beads were used as a counting reference and the flow cytometry was stopped once the number of beads reached a pre-set count.

The number of cells killed=the number of live cells in the no effector cells control – the number of live cells remaining in the experimental group.

Specific lysis=the number of cells killed/the number of live cells in the no effector cells control.

### Proliferation assay

For the estimation of Vγ9Vδ2 T cell proliferation, the cell density in the stimulated PBMC cultures was determined and their volumes were estimated using pipettes. A FACSCalibur (BD Biosciences, San Jose, CA, USA) was used to determine the percentage of Vδ2+CD3+ cells in the initial PBMCs and the stimulated PBMC cultures. The fraction of Vδ2+ cells out of the total CD3+ cells for the donors used in this study are listed in Table S1. Proliferation was defined as the ratio of the number of Vδ2+ T cells on days 10-11 to the number of Vδ2+ T cells on day 0. Relative proliferation was defined as the proliferation of the sample with the proliferation of the IL-2 only control subtracted. The cell numbers were obtained by multiplying the percentage of Vδ2+ T cells by the volume of the sample. The isotype control confirmed that there was no unspecific staining. Data analysis was performed using FlowJo (Tree Star Inc., Ashland, OR, USA).

### Microscopy

To assess cell adherence in the polystyrene wells a Nikon Eclipse TE2000 inverted microscope (Nikon, Tokyo, Japan) equipped with 20× air objective and a Nikon camera was used.

### Statistics

10-logarithmic transformed data were used for the statistical calculations of proliferation. The data were tested for normality using the Shapiro–Wilk test and normality could not be rejected. No substantial heteroscedasticity was found. For the proliferation data where one variable at the time were compared, a paired *t*-test of the means for two biological replicates for each condition was used. The experiments with DMSO were evaluated with a linear statistical model based on 84 observations from 11 donors with freshly prepared/cryopreserved, with/without added DMSO, donors and the experimental date as explanatory factors. The response variable in the model was relative proliferation after 10-logarithmic transformation. For each ratio of the cytotoxicity measurements, an analysis of variance (ANOVA) model was used with donor and HMBPP or donor, HMBPP and DMSO as explanatory factors. For the cytotoxicity methods, 95% confidence intervals were calculated for the different E:T ratios from the means of three biological replicates with a pooled standard deviation. The pooled standard deviation was estimated for each E:T ratio with 27 or 30 observations with a linear regression model with donor, treatment and cell type as explanatory variables and percentage dead cells as the dependent variable. No data were excluded from the statistical analysis. The cut-off values for statistical significance were defined as *P<0.05, **P<0.01 and ***P<0.001.

## Supplementary Material

Supplementary information
