# Supplementary material for: Potentiating Vγ9Vδ2 T cell proliferation and assessing their cytotoxicity towards adherent cancer cells at the single cell level
Source: Biol Open. 2022 Feb 7;11(1):bio059049. doi: 10.1242/bio.059049 (PMC8822357; doi:10.1242/bio.059049)
Supplement: Supplementary information [file biolopen-11-059049-s1.pdf]

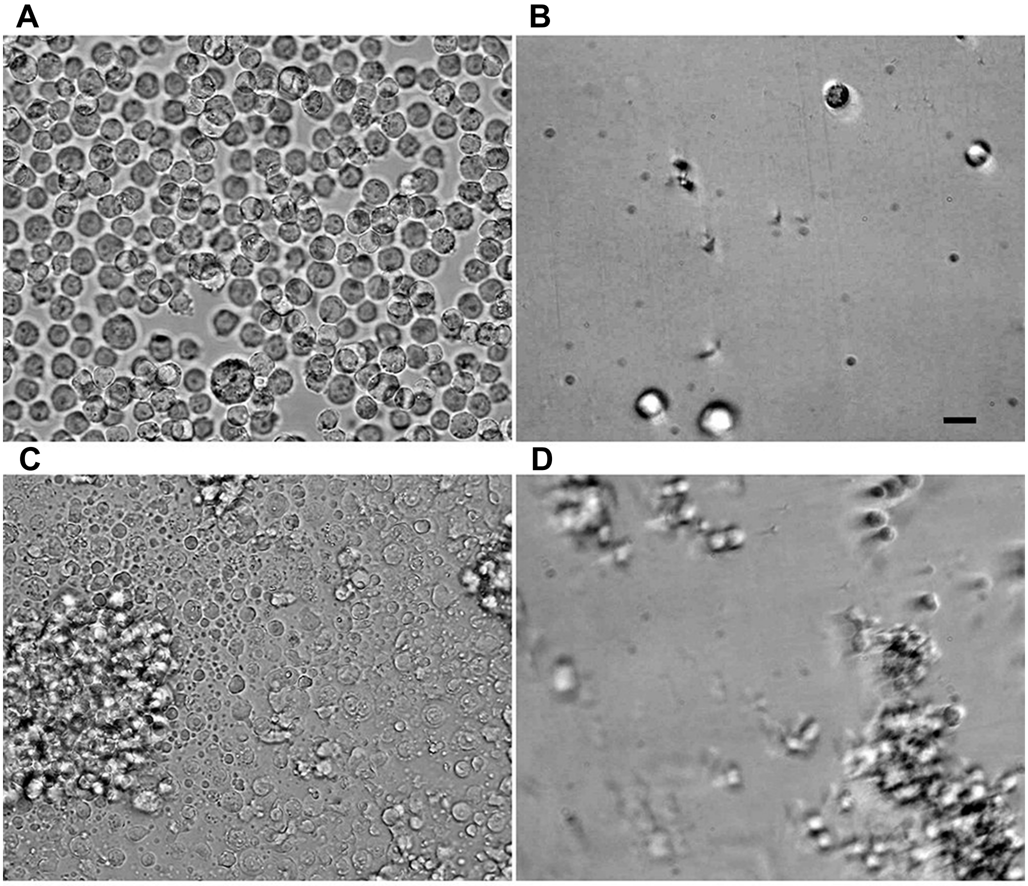

Fig. S1. Neither effector nor target cells adhere to the plastic wells during the cytotoxicity assessment. HT29 cells (A and B) or HT29cells and *in vitro* expanded Vγ9Vδ2 T cells (C and D) were incubated at 37°C in 96-well round bottom polystyrene plates at 5 % CO_2 after which the cells were imaged at the bottom of the wells using a 20X air objective. After 4h most of the HT29 cells were found at the bottom of well (A) as were γδ T cell colonies (C). After suspension with a 100 µl automatic pipette, no cells adhered to the bottom of the wells (B and D). The images in the four panels have been individually contrast stretched. Scale bar 10 µm.

Biology Open • Supplementary information

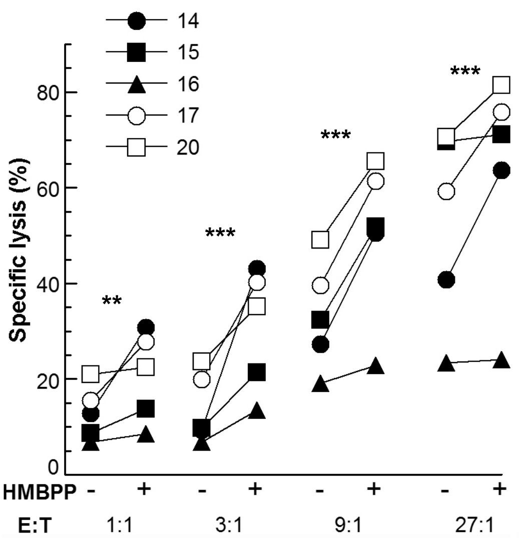

Fig. S2. HMBPP enhances the cytotoxicity of *in vitro* expanded Vγ9Vδ2 T cells towards SW620 cells at low E:T ratios. PBMCs, freshly prepared or cryopreserved, were seeded at $1x10^6$ live cells/ml and stimulated with 80 pM HMBPP. IL-2 (25U/ml) was added days 3, 5, 7 and 9. Total γδ cells (effector cells=E) were purified by negative selection using magnetic beads after 11-12 days of culture. The purified effector cells (E) were incubated with SW620 target (T) cells ± 80 pM HMBPP at different E:T ratios for 4h at 37°C. Specific lysis was determined by flow cytometry analysis. Data shown are means from two biological replicates and stars indicate the statistical significance from an ANOVA for each E:T ratio.
